# Morbidity During the Early Interwar Period (1923-27): A Historical-Epidemiological Study of 15,146 Cases Treated at the “Agios Dimitrios” Hospital in Thessaloniki, Greece

**DOI:** 10.7759/cureus.48004

**Published:** 2023-10-30

**Authors:** Theodoros N Sergentanis, Spyros N Michaleas, Ifigeneia Mpersimi, Vasiliki Traouda, Theodora Psaltopoulou, Marianna Karamanou, Christos D Lionis

**Affiliations:** 1 Department of Public Health Policy, School of Public Health, University of West Attica, Athens, GRC; 2 Department of History of Medicine and Medical Deontology, Medical School, University of Crete, Heraklion, GRC; 3 Department of History of Medicine and Medical Ethics, Medical School, National and Kapodistrian University of Athens (NKUA), Athens, GRC; 4 Department of Management and Administration, General Hospital of Thessaloniki “Agios Dimitrios”, Thessaloniki, GRC; 5 Department of Clinical Therapeutics, “Alexandra” Hospital, Medical School, National and Kapodistrian University of Athens (NKUA), Athens, GRC; 6 Clinic of Social and Family Medicine, Medical School, University of Crete, Heraklion, GRC

**Keywords:** fatality, infectious disease, public health, epidemiology, history of medicine

## Abstract

Background and objective

The period spanning 1923-1927 was a turbulent period in Greek history following the catastrophic defeat of the Greek army in September 1922, known as the “Asia Minor Catastrophe”. The massive settlement of refugees in Thessaloniki, Greece, entailed massive economic, public health, and social challenges. The present historical-epidemiological study aims to evaluate the diseases of individuals hospitalized at the “Agios Dimitrios” Hospital in Thessaloniki during the aforementioned period.

Materials and methods

This study involved 15,146 consecutive patients (January 1923-March 1927) treated at the hospital. Data were collected from the General Hospital of Thessaloniki "Agios Dimitrios” and were manually entered into a pre-coded database. Descriptive statistics were calculated. In addition, the case fatality rates (CFR) were calculated; the respective 95% confidence intervals (CI) were estimated.

Results

The most frequent causes for admission to the hospital were as follows: normal delivery/delivery without disclosed sequalae (n=1915, 12.7%), followed by tuberculosis (n=1514, 10.0%), malaria (n=1438, 9.5%), injuries/falls/fractures (n=1394, 9.2%), pneumonia/pleuritis (n=1010, 6.7%), appendicitis (n=623, 4.1%), dysentery/enterocolitis/typhoid (n=489, 3.2%), salpingitis/salpingo-oophoritis (n=358, 2.4%), soft tissue abscesses (n=309, 2.0%), hernias (n=295, 2.0%), rabies (n=239, 1.6%), metrorrhagia/menorrhagia (n=233, 1.5%), ocular cataract (n=225, 1.5%), postpartum infections/endometritis (n=181, 1.2%), uterine discomfort/uterine pain (n=162, 1.1%), nephritis/uremia (n=157, 1.0%), miscarriage (n=155, 1.0%), skin infections/inflammations excluding abscesses (n=152, 1.0%), otitis/mastoiditis/labyrinthitis (n=96, 0.6%), and peptic ulcer (n=93, 0.6%). Tuberculosis was particularly associated with high CFR (49.5%, 95% CI: 47.2-52.3%), followed by nephritis/uremia (CFR: 37.6%), dysentery/infectious enterocolitis/typhoid (CFR: 24.3%), peptic ulcer (CFR: 22.6%), pneumonia/pleuritis (CFR: 16.1%), postpartum infections/endometritis (CFR: 15.5%).

Conclusions

Infections predominated in the disease spectrum of the hospitalized population. The documented fatality rates were high; poor outcomes may reflect the socioeconomic adversities and limited medical means and resources available at that time.

## Introduction

The catastrophic defeat of the Greek army in Asia Minor during the Turkish-Greek War (1919-1922), known as the “Asia Minor Catastrophe”, occurred in September 1922 and resulted in a huge refugee wave from Turkey to Greece. The Lausanne Peace Treaty (1923) ended the Turkish-Greek War and led to the compulsory exchange of populations between Greece and Turkey based on the criteria of religion [1]. This historical-epidemiological study focuses on this time period. After the end of the Balkan Wars (1912-1913), World War I (1914-1918), the October Revolution (1917), and the Turkish-Greek War (1919-1922), the uprooting of local populations occurred on an unprecedented scale; large populations left their homelands in mass refugee movements. The areas from where these populations moved to Greece included Asia Minor, Serbia, Bulgaria, and the Caucasus [2].

In 1922-1923, thousands of refugees were arriving daily in Greece from the ports of Smyrna, Çesme, Ayvalik, Samsun, Giresun, Istanbul, etc. The refugee wave included Greeks from Asia Minor who were evacuated in panic into the ports of Thessaloniki and Piraeus. It is estimated that more than one million Greeks arrived on the Greek mainland and the islands at that time [3].

The Asia Minor Catastrophe forced the Greek Government to rebuild and reorganize its public institutions [4]. The massive settlement of refugees led to an unprecedented rise in the country’s population in just a few months; it entailed economic, public health, and social upheavals [5]. Typhus, smallpox, dysentery, and tuberculosis epidemics spread in the region of Macedonia and specifically in cities and towns, such as Thessaloniki, Veroia, and Edessa [6]. In the following years, the Greek government and foreign non-governmental organizations tried to help improve the life and health conditions of these refugees; American Red Cross, American Women’s Hospitals, Union de Secours aux Enfants, Swedish Red Cross, Imperial War Relief Fund, British Red Cross, Secours Français aux victimes du Proche-Orient were some of the philanthropic organizations that contributed in this regard [5].

Due to its geopolitical location in Macedonia, Thessaloniki, the second-largest city in Greece, has always been a multicultural crossroad where people of different religious and cultural origins met and coexisted for long periods of time [7]. The hospital of “Agios Dimitrios” in Thessaloniki was built by the Turks in 1902 in an area belonging to the Greek Community; the area had been granted to the Ladies' Charitable Institution in 1871. During its early years, it operated as a hospital for poor people of foreign origin and later as a Municipal Hospital. After the annexation of Thessaloniki by Greece (Treaty of Bucharest, 1913), “Agios Dimitrios” Hospital served as a Municipal Hospital. After the Treaties of Neuilly (1919) and Lausanne (1923), Greek refugees, as well as local inhabitants, were treated at this institution [8,9].

In light of this, the present historical-epidemiological study aimed to evaluate the disease spectrum of individuals hospitalized at the “Agios Dimitrios” Hospital, Thessaloniki, and involves a sizable dataset of 15,146 consecutive patients hospitalized during the period spanning January 1923-March 1927, with a view to shed light on the public health turmoil during that period.

## Materials and methods

Data collection

Data were collected from the General Hospital of Thessaloniki "Agios Dimitrios”. Permission was granted by the Scientific Council of the Hospital (board meeting 4/19-02-2019, topic 12) and the 3rd Health District Directorate of Thessaloniki (board meeting 4/28-02-2019; topic 28). This unpublished archival material comprises the data of 15,146 consecutive patients who were admitted to the “Agios Dimitrios” Hospital from January 01, 1923, to March 31, 1927. The archival material included information about the date of hospitalization, gender, age, religion, profession, marital status, place of birth, place of residence, diagnosis, outcome, and date of discharge or death. Records were manually entered into a pre-coded database so that statistical analysis could be performed.

Quality control of the data entered into the database was performed by double-checking of records by using a random selection of unique codes by two independent reviewers (T.N.S. and S.M.).

Statistical analysis

Data were tabulated and descriptive statistics were appropriately calculated with regard to the study variables [gender, refugee status, diagnosis at hospitalization, age at hospitalization (years), and length of hospitalization in days]. Regarding diagnoses at hospitalization, only conditions involving 30 hospitalized cases or more were presented in the relevant tables. Concerning categorical variables, frequencies and relative frequencies (%) were presented; regarding continuous variables median as well as 25th and 75th percentile were calculated.

Afterward, the case fatality rate (CFR) was calculated for the 20 most frequent conditions documented in the records; the respective 95% confidence intervals (CI) were estimated on the basis of the binomial distribution. Statistical analysis was performed using the STATA/SE version 13 statistical software (Stata Corp., College Station, TX).

## Results

The sociodemographic characteristics of the study subjects, as well as the causes of morbidity, are shown in Table 1.

**Table 1:** Sociodemographic characteristics and causes of morbidity in the study subjects (n=15,146) Only conditions with 30 hospitalized cases or more are shown

Sociodemographic characteristics - categorical variables	N (%)
Gender	
Male	6994 (46.2)
Female	8137 (53.8)
Not available	15 (0.1)
Refugee status - place of origin	
Refugee	8983 (59.3)
Mainland Greek regions	6163 (40.7)
Sociodemographic characteristics - continuous variables	Median (25th-75th percentile)
Age at hospitalization (years)	29 (21-40)
Length of hospitalization (days)	12 (7-23)
Causes of morbidity – infectious/communicable diseases	N (%)
Tuberculosis	1514 (10.0)
Malaria	1438 (9.5)
Pneumonia/pleuritis	1010 (6.7)
Dysentery/infectious enterocolitis/typhoid	489 (3.2)
Salpingitis/salpingo-oophoritis	358 (2.4)
Soft tissue abscesses	309 (2.0)
Rabies	239 (1.6)
Postpartum infections/endometritis	181 (1.2)
Skin infections/inflammations, excluding abscesses	152 (1.0)
Otitis/mastoiditis/labyrinthitis	96 (0.6)
Keratitis/corneal ulcer	74 (0.5)
Lymphadenopathy/lymphadenitis	70 (0.5)
Dacryocystitis	68 (0.5)
Gangrene	53 (0.4)
Urinary bladder infections	53 (0.4)
Phlegmon of the lower limb	52 (0.3)
Septicemia	44 (0.3)
Echinococcus/hydatid cysts	42 (0.3)
Syphilis	38 (0.3)
Tonsillitis/pharyngitis/laryngitis	34 (0.2)
Pyelonephritis	33 (0.2)
Myocarditis	30 (0.2)
Causes of morbidity - non-communicable conditions	N (%)
Normal delivery/delivery without disclosed sequelae	1915 (12.7)
Injuries/falls/fractures	1394 (9.2)
Appendicitis	623 (4.1)
Hernias	295 (2.0)
Metrorrhagia/menorrhagia	233 (1.5)
Ocular cataract	225 (1.5)
Uterine discomfort/uterine pain	162 (1.1)
Nephritis/uremia	157 (1.0)
Miscarriage	155 (1.0)
Peptic ulcer	93 (0.6)
Rheumatism, “rheumatopathy”	90 (0.6)
Lithiasis of the urinary tract	82 (0.5)
Gastroptosis	73 (0.5)
Gastric cancer	68 (0.5)
Poisoning	63 (0.4)
Hemorrhoids	63 (0.4)
Liver cirrhosis	63 (0.4)
Burns	59 (0.4)
Mitral valve disease (stenosis; insufficiency)	55 (0.4)
Ovarian cysts	54 (0.4)
Cholelithiasis	47 (0.3)
Sciatica	47 (0.3)
Arthritis	46 (0.3)
Concussion	45 (0.3)
Soft tissue sarcomas	44 (0.3)
Hyperchlorhydria	43 (0.3)
Chronic bronchitis	38 (0.3)
Vaginal bleeding during pregnancy	37 (0.2)
Breast cancer	37 (0.2)
Hemiplegia	37 (0.2)
Uterine cancer	34 (0.2)
Hemoglobinuria	34 (0.2)
Uterine cervix stenosis	32 (0.2)
Glaucoma	32 (0.2)
Uterine prolapse	30 (0.2)
Tumors of unspecified location	30 (0.2)
Hydrocele	30 (0.2)
Various other infectious or non-infectious conditions, with 29 or less cases	2204 (14.6)

**Figure 1:**
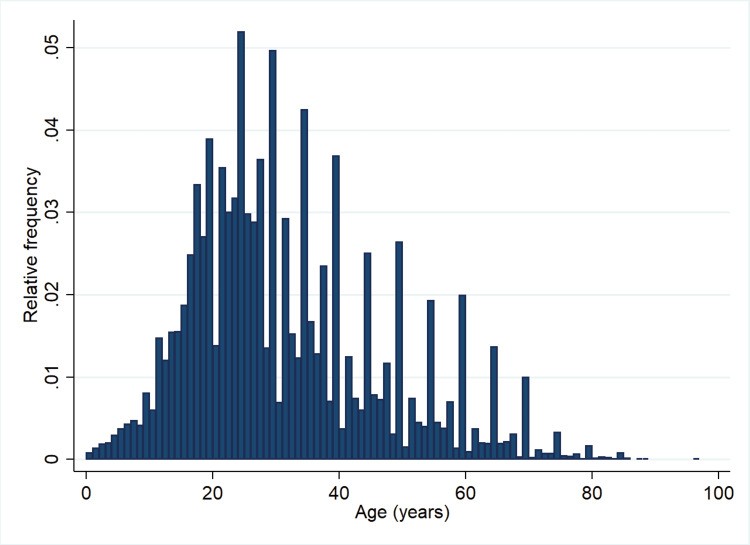
Histogram depicting the age distribution in the hospitalized patients

Of note, 53.7% of the hospitalized patients were females. The majority of the subjects were refugees (59.3%, n=8983). A histogram of the age distribution among the hospitalized patients is shown in Figure 1.

The median age of the cohort was 29 years (25th-75th percentile: 21-40 years); age at hospitalization varied vastly, from newborns to 97 years. The 20 most frequent conditions for admission to the hospital (Table 1) were as follows: normal delivery/delivery without disclosed sequalae (n=1915, 12.7%), followed by tuberculosis (n=1514, 10.0%), malaria (n=1438, 9.5%), injuries/falls/fractures (n=1394, 9.2%), pneumonia/pleuritis (n=1010, 6.7%), appendicitis (n=623, 4.1%), dysentery/enterocolitis/typhoid (n=489, 3.2%), salpingitis/salpingo-oophoritis (n=358, 2.4%), soft tissue abscesses (n=309, 2.0%), hernias (n=295, 2.0%), rabies (n=239, 1.6%), metrorrhagia/menorrhagia (n=233, 1.5%), ocular cataract (n=225, 1.5%), postpartum infections/endometritis (n=181, 1.2%), uterine discomfort/uterine pain (n=162, 1.1%), nephritis/uremia (n=157, 1.0%), miscarriage (n=155, 1.0%), skin infections/inflammations excluding abscesses (n=152, 1.0%), otitis/mastoiditis/labyrinthitis (n=96, 0.6%), and peptic ulcer (n=93, 0.6%).

Other common conditions, in descending order of frequency (Table 1), included rheumatism, “rheumatopathy” (n=90, 0.6%), lithiasis of the urinary tract (n=82, 0.5%), keratitis/corneal ulcer (n=74, 0.5%), gastroptosis (n=73, 0.5%), lymphadenopathy/lymphadenitis (n=70, 0.5%), gastric cancer (n=68, 0.5%), dacryocystitis (n=68, 0.5%), poisoning (n=63, 0.4%), hemorrhoids (n=63, 0.4%), liver cirrhosis (n=63, 0.4%), burns (n=59, 0.4%), mitral valve disease (n=55, 0.4%), ovarian cysts (n=54, 0.4%), gangrene (n=53, 0.4%), urinary bladder infections (n=53, 0.4%), phlegmon of the lower limb (n=52, 0.3%), cholelithiasis (n=47, 0.3%), sciatica (n=47, 0.3%), arthritis (n=46, 0.3%), concussion (n=45, 0.3%), septicemia (n=44, 0.3%), soft tissue sarcomas (n=44, 0.3%), hyperchlorydria (n=43, 0.3%), echinococcus/hydatid cysts (n=42, 0.3%), syphilis (n=38, 0.3%), chronic bronchitis (n=38, 0.3%), vaginal bleeding during pregnancy (n=37, 0.2%), breast cancer (n=37, 0.2%), hemiplegia (n=37, 0.2%), tonsillitis/pharyngitis/laryngitis (n=34, 0.2%), uterine cancer (n=34, 0.2%), hemoglobinuria (n=34, 0.2%), pyelonephritis (n=33, 0.2%), uterine cervix stenosis (n=32, 0.2%), glaucoma (n=32, 0.2%), uterine prolapse (n=30, 0.2%), myocarditis (n=30, 0.2%), tumors of unspecified location (n=30, 0.2%), and hydrocele (n=30, 0.2%).

In-hospital CFRs for the 20 most common conditions in the study are shown in Table 2.

**Table 2:** In-hospital CFR for the 20 most frequent conditions in the study *Cases with missing status regarding the death outcome were excluded from this analysis; ^§^one-sided 97.5% confidence interval due to no observed events CI: confidence interval; CFR: case fatality rate

Diagnosis	CFR*	95% CI
Normal delivery/ delivery without disclosed sequelae	0.5% (9/1906)	0.2-0.9%
Tuberculosis	49.7% (753/1514)	47.2-52.3%
Malaria	6.9% (99/1438)	5.6-8.3%
Injuries/falls/fractures	10.0% (139/1394)	8.4-11.7%
Pneumonia/pleuritis	16.1% (163/1010)	13.9-18.6%
Appendicitis	4.2% (26/623)	2.7-6.1%
Dysentery/infectious enterocolitis/typhoid	24.3% (119/489)	20.6-28.4%
Salpingitis/salpingo-oophoritis	1.4% (5/358)	0.5-3.2%
Soft tissue abscesses	12.3% (38/309)	8.9-16.5%
Hernias	7.8% (23/295)	5.0-11.5%
Rabies	0.8% (2/239)	0.1-3.0%
Metrorrhagia/menorrhagia	0.0% (0/233)	0-1.6%^§^
Ocular cataract	0.0% (0/225)	0-1.6%^§^
Postpartum infections/endometritis	15.5% (28/181)	10.5-21.6%
Uterine discomfort/uterine pain	0.0% (0/162)	0-2.3%^§^
Nephritis/uremia	37.6% (59/157)	30.0-45.7%
Miscarriage	1.9% (3/155)	0.4-5.5%
Skin infections/inflammations, excluding abscesses	5.3% (8/152)	2.3-10.1%
Otitis/mastoiditis/labyrinthitis	9.4% (9/96)	4.3-17.1%
Peptic ulcer	22.6% (21/93)	14.6-32.4%

Tuberculosis was particularly associated with high CFR (49.5%, 95% CI: 47.2-52.3%), followed by nephritis/uremia (CFR=37.6%, 95% CI: 30.0-45.7%), dysentery/infectious enterocolitis/typhoid (CFR: 24.3%, 95% CI: 20.6-28.4%), peptic ulcer (CFR: 22.6%, 95% CI: 14.6-32.4%), pneumonia/pleuritis (CFR: 16.1%, 95% CI: 13.9-18.6%), postpartum infections/endometritis (CFR: 15.5%, 95% CI: 10.5-21.6%), soft tissue abscesses (CFR: 12.3%, 95% CI: 8.9-16.5%), and injuries/falls/fractures (CFR: 10.0%, 95% CI: 8.4-11.7%). Other conditions with CFR exceeding 5% were as follows: otitis/mastoiditis/labyrinthitis (CFR: 9.4%, 95% CI: 4.3-17.1%), hernias (CFR: 7.8%, 95% CI: 5.0-11.5%), malaria (CFR: 6.9%, 95% CI: 5.6-8.3%), and skin infections/inflammations excluding abscesses (CFR: 5.3%, 95% CI: 2.3-10.1%).

## Discussion

This historical epidemiological study portrays the wide spectrum of diseases that affected the refugee and local population of Thessaloniki during the early Interwar Period (1923-27), after the Asia Minor Catastrophe (1922), and involves a sizable dataset of 15,146 consecutive hospitalized patients. Infectious diseases predominated, as tuberculosis, malaria, pneumonia and pleuritis, dysentery/infectious enterocolitis/typhoid, salpingitis/salpingo-oophoritis, soft tissue abscesses, menorrhagia, ocular cataract, postpartum infections and endometritis, skin infections/inflammations and otitis together with its most common complications (mastoiditis and labyrinthitis) were among the 20 most prevalent causes of hospitalization.

Among infections, malaria was found to be an important source of morbidity; the high rate of hospitalization demonstrated that the high prevalence of the disease was due to extensive uncultivated marsh areas in the region of Central Macedonia as well as the climatic conditions that favored the spread of mosquitoes [10]. The results of the present study are in line with our previous epidemiological study based in another smaller town of Macedonia, Northern Greece, during the Interwar Period, namely Veroia, which is located approximately less than 100 km from Thessaloniki. In the Veroia cohort, malaria was the leading cause of hospitalization, accounting for more than half of the hospitalized cohort (52.8%, 8408 of 15,921 cases) [11]. The fact that many refugees originated from contaminated or marshy areas in their homelands such as Amisos of Pontus, Caucasus, and Aydin has also contributed to the high prevalence of malaria [12]. Encountering this situation, the Anti-Malaria Battle was a long-term effort that shaped the foundations of public health policy in Greece, aiming to combat and mitigate the burden of malaria in the country [13]; Constantinos Savvas (1861-1929) [14] and Ioannis Kardamatis (1859-1942) [15] were among the most influential personalities in the nationwide battle against malaria.

The “Agios Dimitrios” Hospital provided obstetric care at that time [9], although during the first half of the 20th century, midwives largely provided peripartum care on an outpatient basis in Greece [16]. Also, the “Agios Dimitrios” Hospital provided ophthalmological care; ocular cataract was among the top 20 causes of hospitalization, accounting for 1.5% of total cases. Interestingly, this pattern contrasts with our previous findings concerning the same time period in the aforementioned, nearby smaller town of Veroia [17]; in the Veroia study, only a minority of cases pertained to ocular conditions. This discrepancy, pointing to a larger representation of ocular conditions in the present study, might signal that patients with ocular cataracts were referred to the “Agios Dimitrios” hospital from various nearby regions, given that this condition necessitated specialized, surgical care [18].

Apart from tracing the most common causes of morbidity during the study period, an interesting aspect of this retrospective epidemiological study pertains to the calculation of in-hospital CFR for the most common conditions, shedding light on the outcomes of patients during that turbulent historical period. Tuberculosis, the second most frequent condition in our study, was associated with the highest CFR, as nearly one in two hospitalized patients passed away due to this condition. Indeed, at that time, several decades before the discovery of isoniazid in 1952, tuberculosis (also known as “phthisis” or “consumption”) was considered the deadly, endemic “White Plague” [19], with considerable mortality documented in the United States, United Kingdom, and worldwide [20,21]. Accordingly, in the present study, pneumonia and pleuritis were associated with particularly high CFR (16.1%). During the Interwar Period in Greece, tuberculosis was envisaged as a social disease, and the Anti-tuberculosis Battle was based on trial detection - isolation - prophylaxis [22,23]. Important milestones included the establishment of the Panhellenic Association Against Tuberculosis in 1901, the First Hellenic Conference Against Tuberculosis in 1909, and the publication of the reference book "The Battle Against Tuberculosis" in 1927 [22].

Peptic ulcer also had a high fatality rate: 22.6% of hospitalized patients died from the condition in the hospital. Peptic ulcer was considered a pandemic at that time; perforations of peptic ulcers were fairly common manifestations [24]. During that period, significant differences between various socioeconomic strata of the society have been documented in other countries, such as the United Kingdom, with the highest mortality rates being noted in lower social classes [25]; such analyses had not been performed in Greece at that time, but it is tempting to conclude that social and nutritional adversities faced by the refugees and local people during that period contributed to the high documented fatality rate due to peptic ulcer.

Other conditions with high fatality rates in this historical-epidemiological study also merit analysis. More than one out of three patients diagnosed with nephritis/uremia died; indeed, at that time, nephrology was merely an emerging field. The term “uremia” was introduced in 1847 by Pierre Adolphe Piorry (1794-1879) [26]; however, effective treatment for uremic comatose patients remained elusive until the development of renal dialysis by the Dutch physician Willem Kolff in 1943 [27]. Dysentery, typhoid, and gastrointestinal infections were also major public health problems in Thessaloniki; the Refugee Settlement Commission had to administer anti-dysentery serum and vaccine at that time [28]. In our study, the triad of dysentery/infectious enterocolitis/typhoid was associated with particularly high fatality, as nearly one out of four hospitalized patients with those conditions passed away during their hospital stay (CFR: 24.3).

The role of this historical study is significant in terms of its focus on public health and society at a crucial period in Greece's history. The hitherto neglected issue of the disease spectrum of the Greek refugees during the Interwar Period is the center of attention. Refugees faced racism and xenophobia on a great scale [29]. The local population was often against their settlement; they often treated them as foreigners coming from Turkey and not as Greek people. Nevertheless, refugees were finally integrated into Greek society and new cultural norms flourished on this crossroad, such as the “rebetiko” music, integrating elements of refugee music into mainland Greece [30]. Refugee waves still hamper our modern world; at the time of drafting this article, the four-million refugee wave that fled Ukraine is a reminder of the need to ensure their dignity and well-being in the realms of public health and policy-making [31].

Despite the various strengths of this study, based on the analysis of a sizable dataset of 15,146 cases, it has a few limitations that need to be addressed. Firstly, we did not find any details regarding the external validity of diagnoses; the analysis was based on the diagnoses declared in the hospital files. Misdiagnosis of cases cannot be ruled out in view of the limitations of medicine at that time; therefore, information bias may have hampered the results. Other limitations pertain to the fact that the recorded CFR pertained only to the hospital stay; as a result, deaths after hospitalization were not reported. Moreover, the data were abstracted from the hospital records; no information on diagnostic procedures implemented for differential diagnosis was available. Accordingly, the hospitalization ratio was not available. Furthermore, details on the administered treatments, surgical or medicinal, were not recorded in the archive, as a rule.

## Conclusions

This study documents the adversities faced by the refugee and local population of Thessaloniki, Greece, during the early Interwar Period. The relevance and novelty of this study pertain to the analysis of morbidity and fatality rates based on previously unpublished archival material, thereby bridging epidemiology and the history of medicine. Infections, namely tuberculosis, malaria, and pneumonia, predominated in the disease spectrum among hospitalized patients. The documented fatality rates were high; poor outcomes may reflect the socioeconomic adversities and limited medical means and resources available at that time.
